# Exploring the relationship between chronic undernutrition and asymptomatic malaria in Ghanaian children

**DOI:** 10.1186/1475-2875-9-39

**Published:** 2010-02-02

**Authors:** Benjamin T Crookston, Stephen C Alder, Isaac Boakye, Ray M Merrill, John H Amuasi, Christina A Porucznik, Joseph B Stanford, Ty T Dickerson, Kirk A Dearden, DeVon C Hale, Justice Sylverken, Bryce S Snow, Alex Osei-Akoto, Daniel Ansong

**Affiliations:** 1 Department of Family and Preventive Medicine, University of Utah, 375 Chipeta Way, Salt Lake City, UT 84108, USA; 2 Komfo Anokye Teaching Hospital, PO Box 1934, Kumasi, Ghana; 3 Department of Health Sciences, Brigham Young University, 229 Richards Building, Provo, UT 84602, USA; 4 Department of Pediatrics, University of Utah, 100 North Mario Cappechi Drive, Primary Children's Medical Center, Salt Lake City, UT 84113, USA; 5 Department of International Health and Center for International Health and Development, Boston University, 801 Massachusetts Avenue, Crosstown Center, 3rd Floor, Boston, MA 02118, USA; 6 Department of Internal Medicine, School of Medicine 4C104, 30 N 1900 E, Salt Lake City, UT 84132, USA; 7 Department of Child Health, School of Medical Sciences, Kwame Nkrumah University of Science and Technology, Kumasi, Ghana

## Abstract

**Background:**

A moderate association has been found between asymptomatic parasitaemia and undernutrition. However, additional investigation using the gold standard for asymptomatic parasitaemia confirmation, polymerase chain reaction (PCR), is needed to validate this association. Anthropometric measurements and blood samples from children less than five years of age in a rural Ghanaian community were used to determine if an association exists between chronic undernutrition and PCR-confirmed cases of asymptomatic malaria.

**Methods:**

This was a descriptive cross-sectional study of 214 children less than five years of age from a community near Kumasi, Ghana. Blood samples and anthropometric measurements from these children were collected during physical examinations conducted in January 2007 by partners of the Barekuma Collaborative Community Development Programme.

**Results:**

Findings from the logistic model predicting the odds of asymptomatic malaria indicate that children who experienced mild, moderate or severe stunting were not more likely to have asymptomatic malaria than children who were not stunted. Children experiencing anaemia had an increased likelihood (OR = 4.15; 95% CI: 1.92, 8.98) of asymptomatic malaria. Similarly, increased spleen size, which was measured by ultrasound, was also associated with asymptomatic malaria (OR = 2.17; 95% CI: 1.44, 3.28). Fast breathing, sex of the child, and age of the child were not significantly associated with the asymptomatic malaria.

**Conclusions:**

No significant association between chronic undernutrition and presence of asymptomatic malaria was found. Children who experience anaemia and children who have splenomegaly are more likely to present asymptomatic malaria. Programmes aimed at addressing malaria should continue to include nutritional components, especially components that address anaemia.

## Background

More than half of child mortality related to major infectious diseases is attributed at least in part to undernutrition [1]. For many infectious diseases, such as acute lower respiratory infections, undernutrition is associated with risk of experiencing the infection in addition to the risk of dying from it [2-5]. In the case of malaria, which causes more than 200 million morbid episodes and more than one million child deaths each year, 57.3% of deaths are attributed to undernutrition [6-10]. Further, undernutrition has been shown to impact both the manifestation of and susceptibility to malaria [7,11].

Not all research, however, agrees that malaria and undernutrition have a synergistic relationship [9,12-14]. For example, several hospital-based studies suggest a protective effect against malaria for children experiencing protein energy malnutrition [15-17]. Other studies have shown similar protective effects against malaria for other forms of undernutrition [18,19]. Most recent research, though, indicates that undernutrition worsens malaria morbidity and mortality [9,11,20-25]. Further, the causal relationship between undernutrition and malaria is a complicated one. On one hand, research shows that undernutrition increases susceptibility to malaria [7,9]. On the other, research indicates that malaria increases the likelihood of a child having poor nutritional outcomes [26-28]. Finally, most studies exploring the relationship between undernutrition and malaria have looked at symptomatic cases of malaria, leaving the relationship between asymptomatic cases of malaria and undernutrition often overlooked.

Malarial infection does not always result in symptomatic or overt disease. Children who experience malarial infection, but who do not exhibit acute symptoms, have asymptomatic parasitaemia, which is common in malaria endemic areas of sub-Saharan Africa, with some areas experiencing parasitaemia prevalence as high as 90% [29-34]. Most research thus far in the area of asymptomatic parasitaemia and undernutrition has pointed to an association between asymptomatic malaria and various measures of undernutrition [12,35-41], though some studies have found no association between undernutrition and asymptomatic malaria [42,43]. More research in this area, particularly research that uses polymerase chain reaction (PCR) testing to confirm parasitaemia in asymptomatic children, is needed.

This study took place in the country of Ghana, where malaria is endemic year round. With a total population of 23 million people, Ghana's health system reported more than 7 million cases of malaria during 2006 and is ranked 8^th ^out of the 19 countries estimated to have 90% of the cases in Africa [10,44]. Nearly one in seven cases required hospitalization and malaria accounts for 44% of all outpatients' visits in Ghana [45]. Further, malaria accounts for 13% of all hospital deaths and 22% of deaths in children less than five years of age in Ghana [44,46]. Finally, *Plasmodium falciparum *infections are estimated to account for approximately 10% of health life-years lost [47].

Anthropometric measurements and blood samples from children less than five years of age in a rural Ghanaian community was evaluated to determine if an association exists between chronic undernutrition (i.e. stunting) and PCR-confirmed cases of asymptomatic malaria. Further, the prevalence of stunting and asymptomatic malaria in addition to other basic physical examination findings is reported.

## Methods

This study uses a cross-sectional design to evaluate the association between stunting and asymptomatic malaria. Data were obtained from physical examinations conducted by partners of the Barekuma Collaborative Community Development Programme (BCCDP). The BCCDP is a collaborative partnership between 20 rural Ghanaian communities near Kumasi, Ghana and researchers from the Komfo Anokye Teaching Hospital (Kumasi, Ghana), the Kwame Nkrumah University of Science and Technology (Kumasi, Ghana), and the University of Utah (Salt Lake City, USA). The BCCDP, which began in 2001, uses a Community Based Participatory Research (CBPR) framework and serves approximately 25,000 people in 20 communities in a rural area approximately 25 kilometers north-west of Kumasi, the second largest city in Ghana [48]. Over the past seven years, partners from the BCCDP have worked with local community leaders to address health issues of concern to the community and partners, such as malaria, schistosomiasis, and diarrhoea. This has been accomplished through physical examination, disease surveillance, census registries, interviews, interventions and community development.

Near the beginning of the collaboration, community partners requested assistance addressing malaria and disease surveillance in the community. Researchers began by first assessing the burden of disease in partner communities. A pilot community was identified in which to conduct physical examination of children to identify common health problems. Data collection was specifically planned to address malaria. The results were intended to be used to inform programme and policy decisions, to test the specificity and sensitivity of a rapid diagnostic test, and to understand the interaction between malaria and other health outcomes in the study area.

Approximately 300 children from Adankwame and surrounding communities were brought by their guardians to the community center to receive a physical examination and to participate in the study. Notice of the study was given to all members of the communities involved and participation was voluntary. Blood samples and anthropometric measurements were available for 214 children less than five years of age.

University of Utah and local Ghanaian pediatricians performed physical examinations on children in the community of Adankwame during January of 2007. Community leaders and researchers encouraged mothers to bring children to the community center to receive the free physical examination. Children were examined on successive days. The physical examination included the collection of standard examination measures, such as age, height, weight, body temperature, pulse, blood pressure, spleen size. Additionally, blood, urine, and stool samples were also collected.

Field workers also gathered information from the child's caregiver on the recent health history of the child including data on recent febrile episodes and malaria symptoms. Binax NOW^® ^Malaria tests were used on-site. Blood smears to check for malaria parasites and complete blood counts were performed at the Komfo Anokye Teaching Hospital malaria laboratory. Additional laboratory work, including malaria PCR of blood smears, was completed at the University of Utah infectious diseases laboratory.

The primary outcome variable is a positive test for asymptomatic malaria parasitaemia. While rapid diagnostic malaria tests were conducted in the field, all blood samples were tested using PCR technology to confirm results from the field. No cases of symptomatic malaria were present in the sample.

Height-for-age z-score (HAZ) was the primary predictor of interest. This measure is based on the international reference standard from Centers for Disease Control and Prevention and World Health Organization. The z-score is calculated through an interpolation function that accounts for sex, age, and height. A child with an HAZ greater than -1.0 standard deviations below the mean on the international reference standard was determined to have no stunting. Mild stunting is height-for-age z-score between -1.0 and -2.0 while moderate stunting is defined as a height-for-age z-score from -2.0 through -3.0. Severe stunting is defined as a height-for-age z-score less than -3.0 standard deviations below the mean on the international reference standard. Other physical examination characteristics examined included fast breathing, anaemia, and spleen size. Fast breathing was defined as greater than 50 breaths per minute in children 2-12 months and greater than 40 breaths per minute for children 12-60 months of age. Anaemia was defined as a child with a haemoglobin count of less than 11.0 (g/dl). Lastly, spleen size was determined through the use of ultrasound.

All data were entered into the computer using Microsoft Excel (version 2003, Seattle, WA, USA). Statistical analyses were conducted using SAS statistical software (version 9.2, Cary, NC, USA). Anthropometric indicators were calculated using a SAS macro designed and made available by the World Health Organization (WHO). This macro uses the WHO International Growth Reference standard. Statistical analyses include a description of the characteristics of respondents. Additionally, percentages used to describe the population and to identify associations between undernutrition and presence of malaria were evaluated using Pearson chi-square tests. Further, prevalence of stunting by age groups was calculated. The relationship between height-for-age z-score and the presence of asymptomatic malaria parasitaemia was analysed using logistic regression. Appropriate confounding variables that were available from physical examination data, such as age and sex, were retained or dropped from the models based on p values (<.1) and conceptual considerations. Regression coefficients and 95% confidence intervals were calculated for retained variables. Interaction among confounders was also examined.

Research ethics approval was obtained in the US from the University of Utah and in Ghana from the Institutional Review Board of Kwame Nkrumah University of Science & Technology School of Medical Sciences and Komfo Anokye Teaching Hospital. Informed consent was obtained from all household heads or guardians of the children enrolled in the study. Participation was voluntary and participants were able to withdraw at any time. Anti-malarials were provided to children who were found to have a positive test for malaria parasitaemia at the time of the physical examination. Children found to need treatment for some other problem were referred to a partnering Ghanaian physician and entered into the Ghana health care system.

## Results

Children were fairly evenly distributed in each age group with four year olds making up the largest single group at 22.9% (Table 1). Nearly half (48.1%) of all children were female. A majority of children experienced no stunting (49.1%) or mild stunting (31.3%). Approximately one-third (31.8%) of children were positive for asymptomatic parasitaemia. Lastly, 29.5% of children were diagnosed as fast breathing while 60.3% were determined to be anaemic.

**Table 1:** Children's characteristics

CHILD CHARACTERISTICS	N = 214
Child age in years (%)	
<1	20.6
1-1.9	21.5
2-2.9	19.2
3-3.9	15.9
4-4.9	22.9
	
Sex, female (%)	48.1
	
Stunting^1 ^(%)	
None	49.1
Mild	31.3
Moderate	13.6
Severe	6.1
	
Asymptomatic Malaria (%)	
Yes	31.8
No	68.2
	
Fast Breathing^2 ^(%)	
Yes	29.5
No	70.5
	
Anaemia^3 ^(%)	
Yes	60.3
No	39.7

^1^Stunting: None = -1 ≤ z-score, Mild = -2 ≤ z-score < -1, Moderate = -3 ≤ z-score < -2, Severe = z-score < -3

^2^Fast Breathing: >50 breaths/minute for children 2-12 months, >40 breaths per minute for children 12-60 months

^3^Anaemia: <11.0 haemoglobin (g/dl)

**Figure 1:**
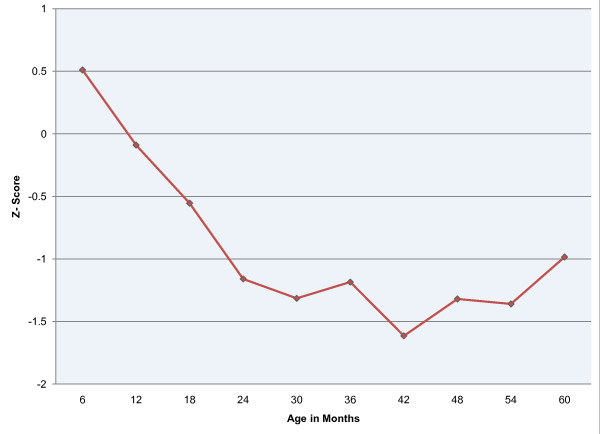
**Median height-for-age z-scores by age**.

The change in HAZ scores generally decreases the older the child was at the time of assessment (Figure 1). Median z-scores begin slightly above the mean reference for younger children and then begin to decline rapidly for each successively older group over the first 24 months of age. During the subsequent 36 months HAZ scores improve only slightly suggesting that children in this area experience little catch-up growth.

The percentage of positive cases of asymptomatic children appears to rise with age (Table 2). For example, of children who were positive for asymptomatic malaria, 10.3% were less than 1 year of age while 30.9% were between four and five years of age. The sex of the child was not significantly associated with asymptomatic malaria (p = 0.423). Further, stunting and fast breathing were not associated with whether or not a child was positive for asymptomatic malaria (p = 0.519 and p = 0.963, respectively). Finally, children who were anaemic were significantly more likely to be positive for asymptomatic malaria (p < 0.001). Specifically, 79.0% of asymptomatic cases were anemic while 51.2% of those who did not have asymptomatic malaria were anaemic.

**Table 2:** Association between children's characteristics and asymptomatic malaria

	Asymptomatic Malaria		
Characteristics	Yes N = 68	No N = 146	p value
Child age in years (%)			0.029
<1	10.3	25.3	
1-1.9	20.6	21.9	
2-2.9	16.2	20.6	
3-3.9	22.1	13.0	
4-4.9	30.9	19.2	
			
Sex (%)			0.423
Female	44.1	50.0	
Male	55.9	50.0	
			
Stunting^1 ^(%)			0.519
None	44.1	51.4	
Mild	38.2	28.1	
Moderate	11.8	14.4	
Severe	5.9	6.2	
			
Fast Breathing^2 ^(%)			0.963
Yes	29.7	29.4	
No	70.3	70.6	
			
Anaemia^3 ^(%)			<0.001
Yes	79.0	51.2	
No	21.0	48.8	

^1^Stunting: None = -1 ≤ z-score, Mild = -2 ≤ z-score < -1, Moderate = -3 ≤ z-score < -2, Severe = z-score < -3

^2^Fast Breathing: >50 breaths/minute for children 2-12 months, >40 breaths per minute for children 12-60 months

^3^Anaemia: <11.0 haemoglobin (g/dl)

Adjusted results for the logistic model predicting the probability of asymptomatic indicate that children who experienced mild, moderate or severe stunting were not more likely to have asymptomatic malaria than children who experienced no stunting (Table 3). Children experiencing anaemia had an increased odds of asymptomatic malaria. Similarly, increased spleen size was also associated with asymptomatic malaria. Fast breathing, sex of the child, and age of the child were not significantly associated with the asymptomatic malaria.

**Table 3:** Relations of stunting, anaemia and spleen size to asymptomatic malaria

Independent Variable	N	Odds Ratio	95% CI
Stunting^1^			
None	74	1.00	--
Mild	60	2.23	0.99, 5.02
Moderate	26	0.56	0.16, 1.69
Severe	12	1.02	0.20, 3.76
			
Anaemia^2^			
No	74	1.00	--
Yes	98	4.15	1.92, 8.98
			
Spleen Size	172	2.17	1.44, 3.28

^1^Stunting: None = -1 ≤ z-score, Mild = -2 ≤ z-score < -1, Moderate = -3 ≤ z-score < -2, Severe = z-score < -3

^2^Anaemia: <11.0 haemoglobin (g/dl)

Variables not retained in the model: fast breathing, sex of child, age of child

## Discussion

These findings show no significant relationship between presence of asymptomatic malaria and stunting, a measure of chronic undernutrition. Children who were mildly stunted, however, appeared to be more likely to be asymptomatic than children who were not stunted, though the difference was not significant. This trend was not found for moderate and severely stunted children. Additionally, there is a strong relationship between anaemia and asymptomatic malaria as well as between spleen size and asymptomatic malaria. Lastly, findings indicate a general decline in median HAZ over the first 24 months of life followed by a leveling off and some subsequent recovery.

While other studies who have found an association between asymptomatic parasitaemia and undernutrition, no such relationship was found in the study population. There could be a number of explanations for these differences. First, several studies found a relationship between asymptomatic parasitaemia and measures of undernutrition that did not include stunting [13,35,39,41]. Further, this study uses PCR, which is considered the gold standard for detection of malaria [49]. Studies that that did find an association with stunting and asymptomatic parasitaemia used a diagnostic method other than PCR [36-38]. Lastly, these results are consistent with studies that found no relationship between malaria infection and undernutrition [40,42,43].

These findings indicating a strong association between presence of asymptomatic malaria and anaemia are consistent with findings from other studies that have linked malaria and anaemia [50-57]. Shankar reports that malaria is "the most significant human parasitic disease" and is a chief cause of anaemia [9]. Other research has shown that a solitary episode of uncomplicated malaria can cause mild anaemia [58]. While these results are not novel in this area, they do validate the data to some extent, thus strengthening the reliability of other findings.

Previous research indicates the key role the spleen plays in protection against malaria [59]. Increased spleen size was shown to be an important predictor of presence of asymptomatic malaria among children in our study. Other studies have shown similar associations with spleen size [53,60]. One study found spleen enlargement in 95-100% of individuals with malaria [59].

This study suffers from some limitations. First, a cross-sectional design was used, which cannot be used to infer causality, but should only used to identify associations. Second, the sample was a convenience sample of children, which may or may not be representative of the entire community or children from the country as a whole. Lastly, only physical examination and brief survey data were available. As a result, statistical models used for this study lack the ability to control for other potential confounders of the relationship between chronic undernutrition and asymptomatic malaria. For example, children from families with favourable economic circumstances may be less likely to be stunted in addition to having reduced exposure to malaria through the purchase and use of insecticide-treated bed nets.

## Conclusions

In conclusion, no significant association between chronic undernutrition as measured by stunting and presence of asymptomatic malaria was found. This result does not suggest that no association exists between undernutrition and clinical malaria. Rather, it appears from these findings that malaria infection is not associated with chronic undernutrition. This is not incongruent with research that indicates that undernutrition may influence the burden of disease related to malaria or that clinical malaria may impact undernutrition [9]. Additionally, results are limited to chronic undernutrition. Measures of acute undernutrition, such as wasting or underweight may be more impacted by and provide more impact on asymptomatic malaria than chronic undernutrition. This relationship could not be explored with data presented here because the study population had very low rates of wasting and underweight. Research exploring the relationship between acute undernutrition and asymptomatic malaria is, therefore, needed. Finally, results do indicate that children who experience anaemia and children who have increased spleen size are more likely to be positive for asymptomatic malaria. Thus, programmes aimed at addressing malaria should continue to include nutritional components, especially components that address anaemia.

## Competing interests

The authors declare that they have no competing interests.

## Authors' contributions

BTC conceived the study, performed the statistical analysis, and drafted the manuscript. SCA participated in the study design, helped develop the BCCDP, assisted in the data collection, and helped to draft the manuscript. IB participated in the data collection and revisions of the manuscript. RMM participated in the study design, assisted in analysis of data, and helped to draft the manuscript. JHA participated in the interpretation of results and the revision of the manuscript. CAP participated in the study design and helped to draft the manuscript. JBS participated in the study design and helped to draft the manuscript. TTD participated in the study design, interpreted the results, and helped to revise the manuscript. KAD participated in the study design, assisted in the data collection, and helped to draft the manuscript. DCH participated in the data collection, helped develop the BCCDP collaboration, and assisted with manuscript revisions. JS participated in the data collection, coordinated the physical examinations, and assisted with manuscript revisions. BSS assisted in the data analysis and manuscript revisions. AOA participated in the data collection, helped coordinate physical examinations, and assisted with manuscript revisions. DA participated in data collection, assisted with the coordination of physical examinations, coordinated the development of the BCCDP, and helped with manuscript revisions. All authors read and approved the final manuscript.
